# A Genetic and Pathologic Study of a DENV2 Clinical Isolate Capable of Inducing Encephalitis and Hematological Disturbances in Immunocompetent Mice

**DOI:** 10.1371/journal.pone.0044984

**Published:** 2012-09-13

**Authors:** Jaime Henrique Amorim, Raíza Sales Pereira Bizerra, Rúbens Prince dos Santos Alves, Maria Elisabete Sbrogio-Almeida, José Eduardo Levi, Margareth Lara Capurro, Luís Carlos de Souza Ferreira

**Affiliations:** 1 Vaccine Development Laboratory, Department of Microbiology, University of São Paulo, Brazil; 2 State University of Santa Cruz, Ilhéus, Brazil; 3 Butantan Institute, São Paulo, Brazil; 4 Institute of Tropical Medicine, University of São Paulo, Brazil; 5 Department of Parasitology, University of São Paulo, Brazil; UC Irvine Medical Center, United States of America

## Abstract

Dengue virus (DENV) is the causative agent of dengue fever (DF), a mosquito-borne illness endemic to tropical and subtropical regions. There is currently no effective drug or vaccine formulation for the prevention of DF and its more severe forms, i.e., dengue hemorrhagic fever (DHF) and dengue shock syndrome (DSS). There are two generally available experimental models for the study of DENV pathogenicity as well as the evaluation of potential vaccine candidates. The first model consists of non-human primates, which do not develop symptoms but rather a transient viremia. Second, mouse-adapted virus strains or immunocompromised mouse lineages are utilized, which display some of the pathological features of the infection observed in humans but may not be relevant to the results with regard to the wild-type original virus strains or mouse lineages. In this study, we describe a genetic and pathological study of a DENV2 clinical isolate, named JHA1, which is naturally capable of infecting and killing Balb/c mice and reproduces some of the symptoms observed in DENV-infected subjects. Sequence analyses demonstrated that the JHA1 isolate belongs to the American genotype group and carries genetic markers previously associated with neurovirulence in mouse-adapted virus strains. The JHA1 strain was lethal to immunocompetent mice following intracranial (i.c.) inoculation with a LD_50_ of approximately 50 PFU. Mice infected with the JHA1 strain lost weight and exhibited general tissue damage and hematological disturbances, with similarity to those symptoms observed in infected humans. In addition, it was demonstrated that the JHA1 strain shares immunological determinants with the DENV2 NGC reference strain, as evaluated by cross-reactivity of anti-envelope glycoprotein (domain III) antibodies. The present results indicate that the JHA1 isolate may be a useful tool in the study of DENV pathogenicity and will help in the evaluation of anti-DENV vaccine formulations as well as potential therapeutic approaches.

## Introduction

Infection with one of the four dengue virus (DENV) serotypes can be asymptomatic or can trigger a wide spectrum of clinical manifestations. The disease may yield symptoms ranging from a mild acute febrile illness, termed dengue fever (DF), to the more severe forms of the disease that include dengue hemorrhagic fever (DHF) and dengue shock syndrome (DSS), characterized by fever, hemorrhage, thrombocytopenia, vascular leakage and viremia that is 10- to 100-fold greater than in DF [1]. The cellular and molecular mechanisms involved in DENV pathogenesis remain, at least in part, elusive. The current hypotheses regarding the mechanisms involved in dengue pathogenicity and the severity of disease symptoms range from dysfunction of the host immune system, with the generation of cross-reactive antibodies and T cells, to platelet depletion, endothelial cell apoptosis and complement activation with damage to host tissues [2]–[7].

The general health state and genetic profile of the host as well as the virulence variability among the DENV strains contribute to the severity of the disease symptoms and development of DHF/DSS [8]–[11]. However, the lack of a more suitable animal model for the study of the disease is a clear drawback to determining DENV pathogenesis and the immunological mechanisms involved in the disease progression or protection [12]. As a corollary, no effective anti-DENV drug or vaccine formulation is presently available for the treatment or prevention of the disease [13].

Humans and mosquitoes represent the only natural hosts for dengue viruses known to date. Some non-human primates are permissive to DENV and elicit specific immune responses, but they develop only transient viremia, without the specific symptoms observed in infected humans [14]–[17]. In addition, for ethical and economic reasons, non-human primates do not represent a sustainable option for the routine research of DENV pathogenicity. Different mouse-based models have been intensively explored as experimental alternatives for the study of DENV pathogenesis and efficacy evaluation of anti-DENV vaccines [12].

Although representing a simple and straightforward tool for the study of DENV pathogenesis, mouse-based DENV infection models have several limitations. First and perhaps most importantly, wild-type DENV strains usually do not infect and kill mice. Thus, the DENV strains used in these studies must be adapted to this new host by serial passages in the brains of suckling mice [18], [19]. During this selective process, different mutations in genes encoding structural and non-structural proteins are selected and confer the ability to replicate and kill the new host of the virus [19]–[23]. However, some of these mutations are expected to change different aspects of the virus physiology, such as cell and tissue tropism as well as the replication rate. Therefore, experimental models based on genetically modified virus strains are expected to have reduced relevance regarding the understanding of viral pathogenicity under natural conditions. Other DENV infection model approaches rely on immunocompromised mouse lineages that lack, for example, genes encoding IFN-γ and IFN-α/β receptors and thus allow the replication of non-adapted DENV strains [24], [25]. Therefore, due to the complex regulatory roles played by these cytokines, the results based on immunodeficient lineages do not necessarily reproduce the conditions expected to be found in immunocompetent mouse lineages or humans [24], [25].

Previous reports indicated that the ability of the mouse-adapted DENV strains to kill immunocompetent mice following administration via the intracranial (i.c.) route is a consequence of the selection of specific mutations during the adaptation process that allow them to replicate and cause encephalitis and death in these animals [18], [20]. In contrast, a previous report of a non-adapted DENV strain capable of infecting and killing immunocompetent mice [26] indicates that the DENV natural genetic plasticity may represent the source of a more suitable DENV infection model for murine hosts. However, the characterization of such a DENV strain as an experimental model for the study of viral pathogenicity and/or the testing of prophylactic and therapeutic interventions has not been fully conducted.

In this study, we characterized a DENV strain recovered from a symptomatic subject that is naturally lethal to immunocompetent mice following i.c. administration. Genetic analysis of the isolate revealed that it is a DENV2 strain grouped within the American genotype. Amino acid sequence analyses demonstrated that the virus strain, named JHA1, has specific polymorphic markers of neurovirulence in mice. The infection with the DENV2 strain induced body weight loss, general tissue damage, altered hematological features indicative of plasma leakage, leucopenia, lymphocytopenia, neutropenia and hemorrhage in Balb/c mice. In addition, the JHA1 strain shares immunological determinants with the NGC strain, a reference DENV2 widely used in the testing of potential anti-dengue vaccine candidates. Collectively, these results indicate that the new DENV2 isolate exhibits several attributes of useful experimental models for studies aimed at understanding viral pathogenicity and vaccine testing.

## Materials and Methods

### Ethics Statement

All handling procedures and experiments involving mice were approved by the Committee for the Ethical Use of Laboratory Animals from the Institute of Biomedical Sciences of the University of São Paulo, in accordance with the recommendations in the guidelines for the care and use of laboratory animals of the National Committee on the Ethics of Research (CONEP). Serum sampling of human beings were approved by the Committee for Ethics in Research Involving Human Beings of the Tropical Medicine Institute of the University of São Paulo and all subjects provided written informed consent.

### Serum Sampling and Viral Isolation

The human serum sample used in the present study was obtained from the Laboratory of Virology-LIM5 collection, at the Tropical Medicine Institute, University of São Paulo. The serum sample was originally collected at the city of Belém (Pará Federal State), in the northern region of Brazil, during the acute phase (day 4 after the symptoms onset) of the disease in a hospitalized patient with dengue fever. The virus was isolated after cultivation in the C6/36 cell lineage [27], as previously described [28], [29].

### Lethality and Propagation of Dengue Virus in Balb/c Mice

Once isolated, the DENV sample was propagated once in C6/36 cells. The cells were cultured in Leibovitz-15 medium (L-15) (Invitrogen, USA) supplemented with 5% fetal bovine serum (FBS) (Gibco, USA). The culture supernatant was collected, divided into aliquots and stored at −80°C or immediately titrated using a plaque assay on LLC-MK2 cells, as previously described [26]. Male Balb/c mice (9 weeks old; *n* = 20) were anesthetized with a mixture of ketamine and xylazine [30] and injected intracranially (i.c.) with 40 µL of L-15 medium supplemented with 5% FBS containing 4,000 plaque-forming units (PFU) or media only (mock group). Mice injected with virus (*n* = 10) were euthanized in the moribund state, 5 days post-infection (p.i.), and the brains were removed and individually macerated in 3 mL of DMEM medium (Gibco, USA) at 4°C. The brain macerates were combined in a 50-mL plastic tube and centrifuged at 405 g for 5 min. The supernatant was harvested, divided into aliquots and stored at −80°C for the virus seed stock. Five randomly chosen aliquots were subsequently titrated by plaque assay on LLC-MK2 cells.

### Nucleotide Sequence and Phylogenetic Analysis

For DENV RNA extraction, 250 µL of an aliquot of the C6/36 cell culture supernatant, collected 4 days after infection with the DENV isolate, was admixed with 750 µL of TRIzol reagent (Invitrogen, USA) and incubated at room temperature for 5 min. A volume of 200 µL of chloroform was added to the initial mixture and incubated for 15 min at room temperature. The mixture was centrifuged at 20,000 g for 10 min at 8°C, and the aqueous phase was collected, added to 500 µL of isopropanol and incubated for 10 min at room temperature. The tube was again centrifuged at 20,000 g for 10 min at 8°C, the supernatant was removed, and the pellet was washed with 75% ethanol (v/v). The pellet was air dried and finally suspended in 20 µL of DEPC-treated water [31]. The extracted RNA was stored at −80°C or immediately subjected to reverse transcription-polymerase chain reaction (RT-PCR) using a specific primer set, according to the manufacturer’s instructions (SuperScript III First-Strand Synthesis SuperMix, Invitrogen, USA). For the amplification reaction, the sense primer used was 5′-GGAATGTCATACTCTAT-3′, and the anti-sense primer was 5′-TTACGATAGAACTTCCTTTCTTA-3′. The amplified sequence encompassed nucleotides 1822 to 3477 of the virus genome, which includes the beginning of domain III of the envelope glycoprotein (EIII) coding sequence to the end of the NS1 coding sequence. The amplified band was purified with the Ilustra™ GFX™ PCR DNA and Gel Band Purification Kit (GE Healthcare Life Sciences, USA) and was directly used in the sequencing reactions. The sample was sequenced four times in both orientations (MegaBACE Sequencer, GE Healthcare, USA). The nucleotide sequence obtained was submitted to GenBank (accession number JQ686088) and aligned with other DENV- and yellow fever virus-equivalent nucleotide sequences using the ClustalW tool. The alignments were used to construct a midpoint-rooted phylogenetic tree by the neighbor-joining method using the Tamura Nei model, implemented by the software MEGA 5.05, with 1,000 bootstrap replicates [32], [33]. Amino acid polymorphism analyses were performed via alignment of the inferred amino acid sequence with sequences from strains of the same genotype, i.e., the India/1957 and Indonesia/1977 strains (GenBank accession numbers FJ538927 and GQ398257, respectively), and the New Guinea C (NGC) strain, which is a reference virus strain selected for neurovirulence in mice (GenBank accession number M29095). The markers for mouse neurovirulence were searched according to a previous report [20]. The comparisons were performed with the amino acid sequences corresponding to part of the envelope glycoprotein (domain III, the adjacent stem-anchor region and the NS1 signal peptide) of the JHA1, India/1957 and Indonesia/1977 strains and with the entire envelope glycoprotein sequence of the NGC strain. Similar analyses were performed with the complete amino acid sequence of the mature NS1 protein of the four DENV2 strains.

### Determination of the LD_50_ in Mice and Pathological Symptoms Induced by the JHA1 Isolate

Male Balb/c mice (9 weeks old) were divided into seven groups (*n* = 10), which were administered i.c. viral loads containing 25, 50, 100, 150, 200 or 300 PFU diluted to a final volume of 40 µL with DMEM. A mock group was injected with DMEM alone. The animals were monitored daily for mortality and changes in body weight, which were recorded over a period of 21 days. The body weight loss was daily calculated for each animal until the day of death or the conclusion of the monitoring period and represented as the percentage of the final weight compared to the initial weight. Seven days post injection (p.i.), the animals were bled via the retro-orbital plexus for the individual determination of hematocrit and lactate dehydrogenase (LDH) levels. For hematocrit determination, heparinized microcapillary tubes (Precision Glass Line, CRAL, Brazil) were filled with blood samples, centrifuged at 4,000 g for 5 min and properly positioned in a packed cell volume table for hematocrit scoring [34], [35]. LDH levels were determined with an analytical kit, as recommended by the supplier (Laborclin, Brazil).

### Lethal Infection with the JHA1 Isolate and Histopathological Analyses

To evaluate the histopathological effects elicited by the JHA1 isolate, 9-week-old male Balb/c mice (*n* = 12) were injected i.c. with 300 PFU of the viral stock. Mice were bled via the retro-orbital plexus (*n* = 6) on days 2, 4, 6 and 7 p.i. for individual serum sampling. The samples were used to test the reactivity of antibodies to recombinant NS1 and EIII proteins (days 2, 4, 6 and 7 p.i.) and INF-γ production (day 7 p.i.). For blood cell analyses, the animals were bled on days 4, 7 and 8 p.i. (*n* = 6), and the blood samples were transferred to microtubes containing EDTA (0.375 M). On the day 8 p.i. (moribund state), the animals were euthanized, and the brains, spleens, lungs, kidneys, liver, colon and bone marrow were removed and individually macerated in DMEM at 4°C (3 mL for each brain, liver, couple of lungs or kidneys, and individual colon; 2 mL for each spleen; and 0.5 mL for each bone marrow). The macerates were transferred to 15-mL plastic tubes and centrifuged for 5 min at 405 g. The virus concentrations in the supernatants were determined using a plaque assay on LLC-MK2 cells. For histological studies, the mice were euthanized (*n* = 3), and the brains were fixed with 10% formaldehyde and processed and stained with hematoxylin and eosin.

### IgG and Cytokine ELISA

Mice sera were tested individually for the presence of NS1 and EIII-specific antibodies by ELISA, as previously described [36]. Briefly, MaxiSorp plates (Nunc, Denmark) were coated with 0.2 µg per well of the recombinant NS1 or EIII proteins (based on the genome sequence of the NGC virus strain) in 100 µL PBS and blocked for 1 h at 37°C with 5% skim milk in 0.05% Tween-20/PBS (PBST). The serum samples were serially diluted and added to wells previously washed with PBST. After 1 h at room temperature, the plates were washed with PBST and incubated with goat anti-mouse IgG conjugated with horseradish peroxidase (Southern Biotechnology, USA) for 1 h at room temperature. The reactions were measured at A_492nm_ with ortho-phenylenediamine dihydrochloride (Sigma-Aldrich, USA) and H_2_O_2_ as a substrate after the addition of a 2 N H_2_SO_4_ stop solution. The titers were established as the reciprocal of serum dilutions that yielded an absorbance two-fold higher than the SD values of the respective mock-infected animals. The serum samples collected on day 7 p.i. were tested individually using an INF-γ, IL-1β or TNF-α ELISA, according to the manufacturer’s instructions (BD Bioscience, USA).

### Blood Cell Analyses

Whole blood samples were used to evaluate six hematological parameters: red blood cell (RBC) and white blood cell (WBC) counts, hematocrit (HTC), platelet number (PTL) and lymphocyte (LYM) and neutrophil (NEU) differentiation. RBC and WBC counts were performed using a Neubauer chamber, HCT was determined as described above, PLT numbers were determined according to Fonio’s method, and NEU and LYM differentiation was performed visually using a phase-contrast microscope [34], [35] (Eclipse E200 model, Nikon, Japan). At the day 7 p.i., mouse plasma were used to perform the prothrombin time test according to the kit manufacturer’s instructions (Bioclin, Brazil).

### Determination of Cross-reacting Epitopes Shared by the Envelope Proteins of the JHA1 and NGC Strains

The immunological relationship between the JHA1 and NGC strains was demonstrated by the labeling of infected cells and virus-neutralization assays performed with antibodies raised in mice immunized with domain III of the recombinant envelope (EIII) protein derived from the NGC strain. Anti-EIII antibodies were obtained in Balb/c mice subjected to an immunization regimen of three doses administered subcutaneously (s.c.) (days 0, 14 and 28) and containing 10 µg of the recombinant EIII combined with complete Freund’s adjuvant (50% v/v) (first dose) and incomplete Freund’s adjuvant in the subsequent injections. The serum samples were pooled, titered with the recombinant protein (final reverse titer of 900,000) and used in JHA1-infected mammalian cells. Sterile microscope cover slips were positioned in wells of Nunc 6-well plates containing 10^5^ cells/well (LLC-MK2) in DMEM supplemented with 5% FBS. After incubation at 37°C with 5% CO_2_ for 12 h, the cells were gently washed with sterile PBS (pH 7), infected with JHA1 and suspended in DMEM at a multiplicity of infection of 1 (MOI = 1) for 1 h at 37°C with 5% CO_2_. The cells were incubated under the same conditions for 96 h and then fixed with 4% paraformaldehyde for 20 min at 4°C, permeabilized with saponin and treated with the anti-EIII serum or serum from non-immune mice for 1 h at room temperature. Subsequently, the cells were treated with goat anti-mouse IgG conjugated to FITC (Invitrogen, USA) and DAPI (Thermo Scientific, USA). The samples were analyzed using an immunofluorescence microscope (Asiovert S100, Zeiss, Germany).

The serum samples were also tested for virus neutralization activity using the JHA1 isolate in plaque assays with the LLC-MK2 cell line, as previously described [37]. In brief, viral stock aliquots were diluted to yield 40 plaques per well (in 6-well plates). The serum pools from immunized or non-immune mice were inactivated (56°C for 30 min), and serial two-fold dilutions beginning at a 1∶2 dilution were added to the virus suspension in a final volume of 400 µL and incubated at 37°C for 1 h. The incubation mixtures were seeded with 2×10^5^ LLC-MK2 cells and incubated for 1 h at 37°C with 5% CO_2_. After incubation, the cells were covered with a mixture containing E199 medium, 1% carboxymethyl cellulose and 2% FBS and incubated at 37°C with 5% CO_2_ for 7 days for viral plaque formation.

The examination of cross-reactive epitopes shared by the NGC and JHA1 strains was also performed with the PRED^BALB/C^ program that predicted the specific class I and II MHC epitopes for Balb/c mice [38]. The epitopes with high scores were compared in both DENV2 strains. The experimentally determined MHC-I-restricted CD8^+^ T cell-specific NS1 epitope (AGPWHLGKL) was also compared in both virus strains [39], [40].

### Statistical Analyses

Statistical analyses were performed using a Student’s *t*-paired test or ANOVA and a subsequent Bonferroni’s multiple comparison test. Statistical significance was set as *p*<0.05.

## Results

### Genetic Study of the DENV JHA1 Strain

**Figure 1:**
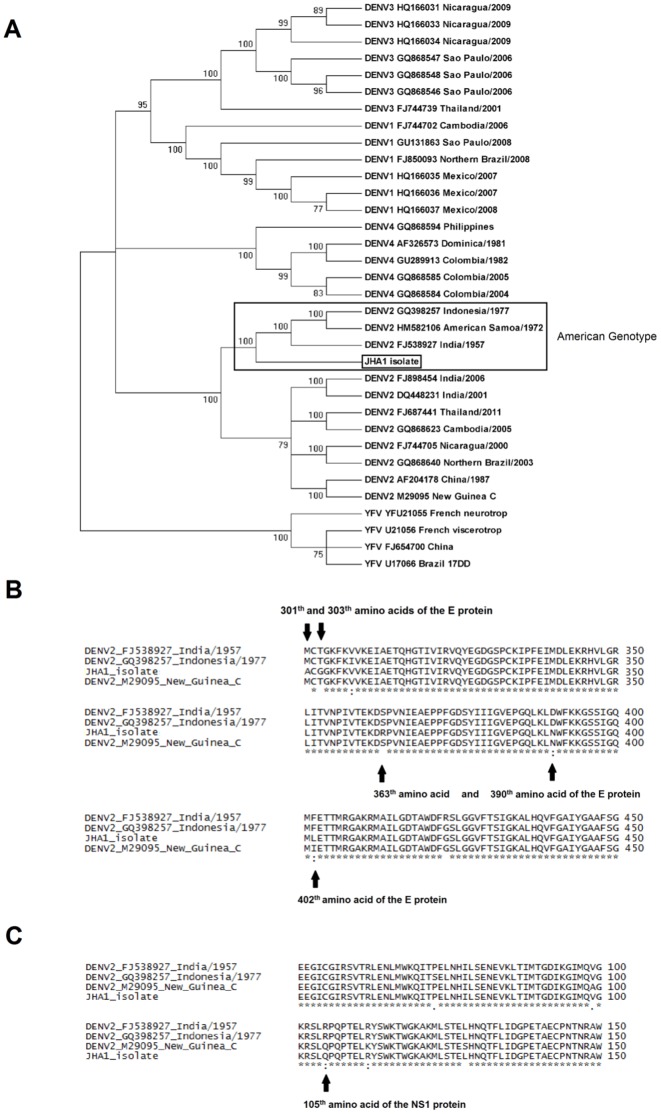
Genetic study of the JHA1 DENV2 strain recovered from a symptomatic subject. (A) Phylogenetic tree showing that the JHA1 strain (indicated by the rectangle) is grouped within the type 2 dengue viruses based on nucleotide sequences encompassing the envelope protein (domain III of the E protein) and the NS1 protein. The JHA1 strain was clustered in the American genotype (India/1957, American Samoa/1972 and Indonesia/1977) strains. Numbers at the nodes are bootstrap values (1000 replicates). DENV sequences were retrieved from GenBank as identified on the right side of the figure. In most cases, the sequences were also identified by the virus isolation area and the year of isolation. (B) Genetic analyses of polymorphic sites within the main envelope (E) protein sequence of the JHA1 strain were performed with the NGC strain, which served as the model for neurovirulence in mice, and the India/1957 and Indonesia/1977 strains, which are unable to infect mice. One amino acid substitution in the JHA1 strain was identical (position 390) and another was similar (position 402) to those observed at the same positions of the NGC strain but not in the other DENV2 strains. Other mutations at positions 301, 303 and 363 of the JHA1 E protein are unique to the JHA1 strain. (C) Genetic analysis of polymorphic sites of the deduced NS1 amino acid sequence. The amino acid replacement at position 105 was found in the NGC strain but not in the other DENV2 strains.

Sequencing of the genes encoding the E (domain III, stem-anchor region and NS1 signal peptide) and NS1 proteins of the JHA1 strain, ranging from nucleotides 1822 to 3477 of the virus genome, and phylogenetic analyses with orthologous nucleotide sequences of different DENV and yellow fever virus (YFV) strains available at the Genbank demonstrated that the strain was a type 2 dengue virus that lies within the American genotype (Figure 1A). Comparison of the amino acid sequences of the JHA1 strain with other DENV2 strains, including the mouse-adapted neurovirulent NGC strain, revealed 23 polymorphic sites (Figure S1). Among these, three polymorphic sites were located on sites previously shown to be involved in neurovirulence in mice [20]. The replacements of an aspartic acid (D) with an asparagine (N) and a phenylalanine (F) with a leucin (L) at positions 390 and 402 of the envelope glycoprotein (E protein), respectively, as well as the replacement of an arginine (R) with a glutamine (Q) at position 105 of the NS1 protein, were ascribed to neurovirulence in mice. These replacements were present in the NGC strain but absent in the other two DENV2 strains isolated from human subjects (Figures 1B, 1C and Table S1). Additional unique polymorphic sites in the E protein sequence of the JHA1 strain were detected, including the replacement of a methyonine (M) with an alanine (A) at position 301, the replacement of a threonine (T) with a glycine (G) at position 303 (see Figure S1) and the replacement of a serine (S) with an arginine (R) at position 363 (see Fig. 1B). Collectively, the sequence analyses indicated that the JHA1 strain would naturally possess features of the NGC strain, particularly regarding neurovirulence in mice.

### The DENV2 JHA1 Strain is Lethal in Immunocompetent Adult Mice

**Figure 2:**
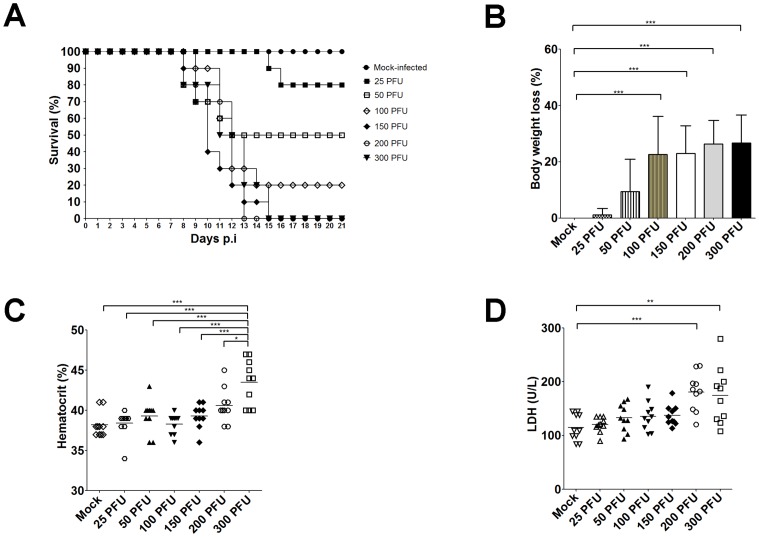
The JHA1 isolate is lethal to immunocompetent mice and causes tissue damage. (A) Survival curves of male Balb/c mice infected i.c. with different viral loads (25 to 300 PFU) in a final volume of 40 µL. Mice were monitored for 21 days after the challenge. (B) Body weight loss in mice infected with the JHA1 strain. The body weights of infected and mock-infected animals were monitored daily, and the differences between the initial and final measurements were calculated and are presented as percentages. (C) Hematocrit determination in mice infected with the JHA1 strain. The animals were bled on the seventh day after the challenge. (D) Serum LDH levels in JHA1-infected mice. The serum samples were collected 7 days after the challenge. Statistically significant differences were determined with the ANOVA test and a subsequent Bonferroni’s multiple comparison test. Statistically significant differences are indicated with asterisks: *, p<0.05; **, p<0.01; and ***, p<0.001. Data are representative of three independent experiments.

After isolation, the JHA1 strain was propagated in the C3/36 cell line and tested for lethality in adult Balb/c mice following i.c. administration. Under these conditions, all animals inoculated with 4,000 PFU of the JHA1 died 5 days later. Subsequent experiments performed to determine the minimum lethal dose showed that the JHA1 strain had an estimated LD_50_ of 50 PFU, while the smallest tested dose (25 PFU) caused a final lethality of 20% under the test conditions (Fig. 2A). Mice infected with 100, 150, 200 or 300 PFU died between 8 and 15 days after challenge and exhibited statistically significant body weight loss compared with the mock-treated animals (Figure 2B). Mice infected with lethal virus loads had morbidity signs (hind limb paralysis and distorted spinal cords) within 24 h before dying; however, morbidity signals were not detected among survivors (data not shown). In addition, the hematocrit values of mice infected with 300 PFU showed a significant increase seven days after challenge when compared with the control group and mice infected with lower viral loads (Figure 2C). Moreover, the serum LDH levels were increased in mice infected with 200 and 300 PFU in comparison to mock-infected animals, suggestive of general tissue damage (Figure 2D). Virus obtained from infected mice were further submitted to sequencing of the genes encoding the EIII, stem-anchor region and NS1 protein and no acquired mutations could be detected with regard to the parental strain (data not shown). Together, these results indicate that the JHA1 strain can kill adult Balb/c mice infected via the i.c. route by causing general tissue injury, including hematological disturbances indicative of plasma leakage.

### Hematological Alterations and Brain Damage in Mice Infected with the JHA1 Strain

**Figure 3:**
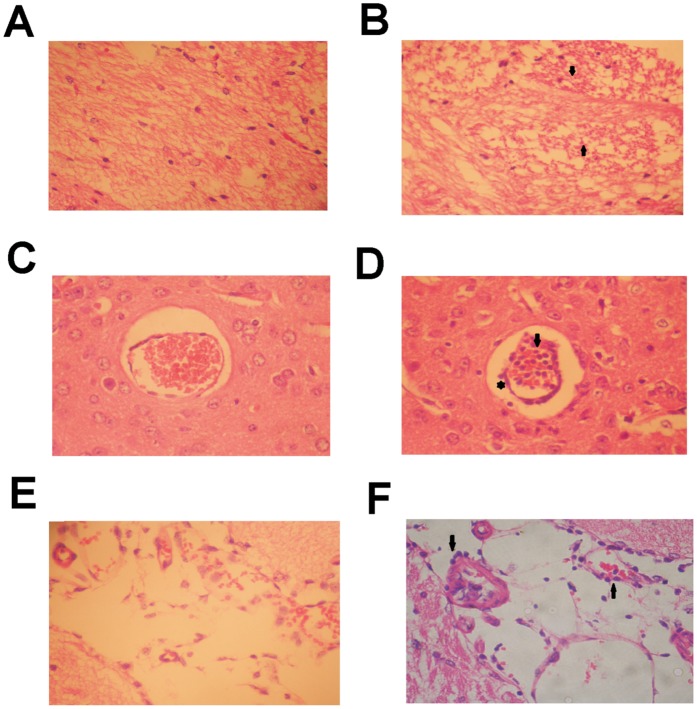
Pathological brain tissue alterations in mice infected with the JHA1 strain. (A) Mock-treated mice with preserved brain parenchyma with typical cellular components. (B) Brain tissue of JHA1-infected mice showing a gliosis scar, in which dead neurons were replaced by astrocytes. The gliosis scar is indicated by the arrows. (C) Brain blood vessel (transverse view) in mock-treated mice. (D) Brain blood vessel in JHA1-infected mice filled with mononuclear cells (arrow). Some of the cells are observed outside the endothelial epithelium (asterisk) and are present in the brain parenchyma (arrowheads). (E) Brain blood vessel (longitudinal view) in mock-treated mice. (F) Brain blood vessel of JHA1-infected mice with infiltration of mononuclear cells (arrowheads). Infected and mock-infected mice were euthanized on day 8 p.i. Brain tissue samples were fixed with 1% formaldehyde, processed and stained with hematoxylin and eosin. Magnification: 400x. Images are representative of three independent experiments.

To investigate the hematological disturbances detected in mice infected with the JHA1 isolate, the animals were inoculated i.c. with 300 PFU (6×LD_50_), and the number of red cells, white cells, and platelets, as well as the hematocrit values, were monitored. As shown in Table 1, no significant cellular parameter was altered on day 4 p.i. However, a clear increase in the hematocrit values and a sharp decrease in the number of white blood cells were observed in JHA1-infected mice 7 days after the challenge. One day later, there was a sharp decrease in the hematocrit values and red blood cell count in virus-infected animals. At this time point, most of the animals were in a moribund state and had begun to die (Table 1). The numbers of lymphocytes and neutrophils were also reduced on days 7 and 8 p.i., but no alteration in the platelet numbers could be detected in mice infected with the JHA1 strain. In contrast, a significant alteration in the coagulation pattern, measured by the prothrombin-dependent coagulation time, was detected in JHA1-infected mice. The prothrombin time was increased by approximately ten-times in mice infected with the JHA1 strain with regard to mock-treated mice, an indication that the coagulation function was drastically impaired among infected mice (Table 1). In addition to the hematological disturbances, the JHA1 strain resulted in brain tissue damage. As shown in Figure 3A, extensive gliosis scars were observed in brain tissue samples collected from mice infected with the JHA1 strain. Moreover, mononuclear cells were detected in the blood vessels and the surrounding brain parenchyma but were not observed in samples collected from the mock-treated animals (Figures 3D and 3F). No apparent spleen or liver damage could be detected in mice infected i.c. with the JHA1 strain (data not shown). These results indicate that the JHA1 strain kills mice mainly via encephalitis with extensive tissue damage.

**Table 1:** Hematological alterations detected in mice infected with the JHA1 strain^a^.

Hematological	Day 4 p.i.		Day 7 p.i.		Day 8 p.i.^c^	
Parameters^b^	Mock-infected	Infected	Mock-infected	Infected	Mock-infected	Infected
**HCT** d	40.3±1.6	39.3±1.2	40.8±1.9	44.5* ±2.0	40.6±1.7	30.0*** ±2.0
**RBC**	7.6±3.0	7.9±1.2	8.7±0.7	11.1±3.3	8.0±0.6	5.8*** ±1
**WBC**	9.0±1.9	5.8±3.2	11.0±0.5	2.4*** ±0.6	8.4±1.1	2.5*** ±0.6
**LYM**	5.8±2.4	4.6±2.9	8.3±0.8	1.7*** ±0.4	7.9±0.8	1.5*** ±0.5
**NEU**	2.0±0.5	1.0±0.8	1.7±0.2	0.6*** ±0.2	1.2±0.1	0.5*** ±0.2
**PLT**	1.2±0.1	1.2±0.1	1.3±0.2	1.3±0.2	1.2±0.1	1.2±0.2
**PT** e			19.5±5.4	217.2±115**		

a Male Balb/c mice (n = 12) were inoculated i.c. with 300 PFU of the JHA1 strain or mock-treated. Mice were bled via the retro-orbital plexus on days 4, 7 and 8 p.i. for hematological analyses.

b Blood samples were processed to determine the concentration of red blood cells (RBCs), white blood cells (WBCs), lymphocytes (LYMs), neutrophils (NEUs), and platelets (PLTs). WBC, NEU, LYM and PLT counts are expressed as 10^3^ cells/µL. RBC counts are expressed as 10^6^ cells/µl.

c On day 8 p.i., the animals had morbidity signals, such as hind limb paralysis and spine cord curvature, which was indicative of a moribund state.

d Hematocrit (HCT) values are given as percentages (%) of packed cell volume. Data are expressed as the mean ± SD of individual measurements. Comparisons between mock-treated and virus-infected mice were performed by t-paired tests on days 4, 7 and 8 p.i., with statistical significance set as p<0.05. (*) p<0.05; (**) p<0.01; (***) p<0.001. The results are based on one representative experiment of three independently performed experiments yielding similar results.

e The prothrombin times (PT) were individually measured on day 7 p.i. both in mock-treated and virus infected mice, in order to access coagulation mechanism integrity. Values are given in seconds. A t-paired test was performed, with statistical significance set as p<0.05. (*) p<0.05; (**) p<0.01; (***) p<0.001 regarding mock-treated and infected groups.

### Replication and Induction of Inflammatory and Adaptive Immune Responses in JHA1-infected Mice

**Figure 4:**
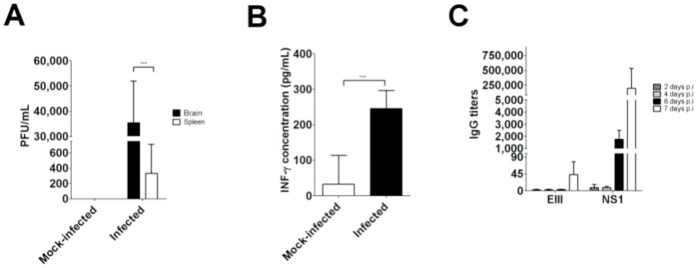
Determination of viable virus particles and induction of inflammatory and adaptive immune responses in mice infected with the JHA1 strain. (A) Replication of the JHA1 strain in brain and spleen tissues. Mice infected i.c. with 300 PFU were euthanized on day 7 p.i., and both the brains and spleens were removed and processed as described in the Methods section. (B) Quantification of INF-γ levels in the serum samples of mice infected with the JHA1 strain. The serum samples were collected seven days after the challenge. (C) Virus-specific serum IgG responses induced in mice infected with the JHA1 strain. The serum samples collected on days 2, 4, 6 and 7 p.i. were tested individually for the presence of NS1- or EIII-specific antibodies by ELISA. Background values detected in serum samples collected from mock-treated animals were reduced from those measured among virus-infected mice.

To determine whether the JHA1 strain remained viable in the tissues of infected mice, organ (brain, spleen and liver) and blood samples were collected 7 days after the challenge with 300 PFU. Viable virus particles were detected in the brains of infected mice (mean number of 70,832±33,228 PFU/brain). The virus was also detected in the spleens of infected mice, albeit at lower levels (333±376 PFU/spleen). No viable virus particles could be recovered from the blood, liver, lungs, kidneys, colon and bone marrow samples. The production of IFN-γ in the serum of virus-infected animals was also determined to be an indication of an acute inflammatory reaction. As indicated in Figure 4B, the IFN-γ serum concentration was significantly enhanced in mice infected with the JHA1 strain compared with the mock-treated animals. But IL-1β and TNF-α were not detected in mice sera of both groups (data not shown). In addition, a dramatic increase in the anti-NS1 serum IgG titers and, to a lesser degree, the anti-EIII IgG levels was detected in the serum of JHA1-infected mice (Figure 4C). Neither NS1 nor EIII-specific antibody responses were detected in mock-infected mice, as evaluated both by ELISA and Western blot (Figure 4C and data not shown). Collectively, these results indicate that the JHA1 strain replicates and induces acute inflammatory reactions and early adaptive immune responses in adult mice infected via the i.c. route.

### The JHA1 Strain Shares Immunological Determinants with the NGC Strain

**Figure 5:**
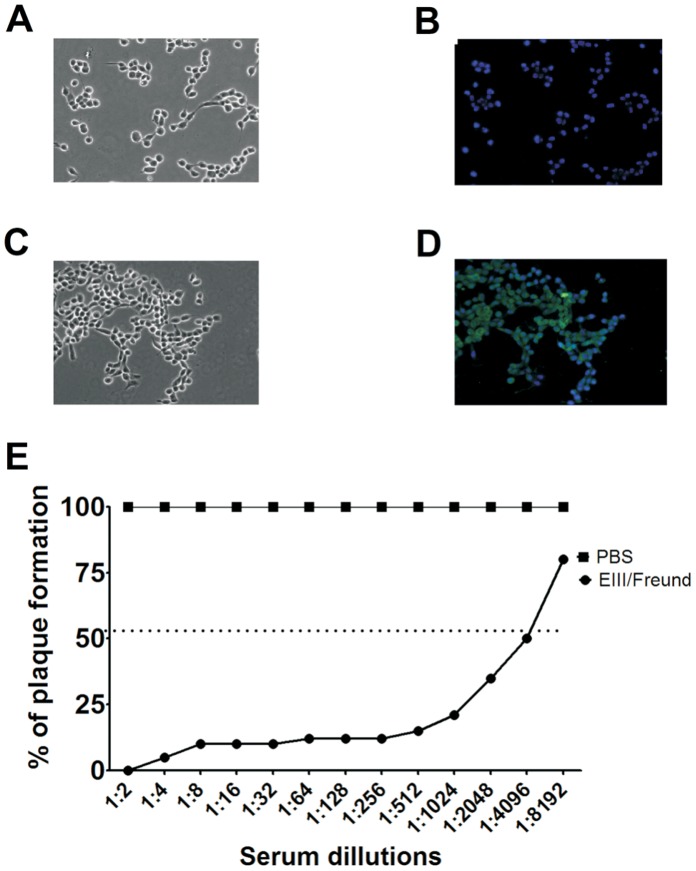
The JHA1 and NGC strains share immunological determinants within the major envelope protein. LLC-MK2 cells were infected with the JHA1 strain and probed with a serum pool raised in mice immunized with EIII derived from the NGC strain or in non-immune animals. (A) Phase-contrast microscopy of the infected cells probed with serum from sham-treated mice. (B) Immunofluorescence microscopy of the infected cells probed with serum from sham-treated mice. The photograph was merged with the same field observed using phase-contrast microscopy. (C) Phase-contrast microscopy of infected cells probed with the anti-EIII serum pool. (D) Immunofluorescence microscopy of infected cells probed with the serum pool of mice immunized with the EIII protein derived from the NGC strain JHA1. The picture was merged with the same field observed in phase contrast. (E) Virus neutralization assay performed with the serum from JHA1-infected mice immunized with the EIII protein derived from the NGC strain. Aliquots containing 40 PFU were incubated with different dilutions of the anti-EIII serum pool (ranging from 1∶2 to 1∶8,192) for 30 min and subsequently transferred to wells with LLC-MK2 cells. One week later, the number of virus plaques was counted. Magnification: 400x. Images are representative of three independent experiments.

In contrast to the DENV2 NGC strain, a viral strain adapted for neurovirulence in mice [41], our results demonstrated that the JHA1 strain was capable of replicating and killing adult Balb/c mice infected via the i.c. route without the need of adaptation steps for the new host. As the NGC strain represents a reference strain both in studies focusing on DENV pathogenesis and the evaluation of anti-dengue vaccine efficacy, we determined the extent of common immunological determinants between the NGC and JHA1 DENV2 strains. Computational analysis performed with the PRED^BALB/C^ program indicated that the class I and II MHC-restricted epitopes of the E glycoprotein (domain III and stem-anchor region) and the NS1 proteins were identical in both virus strains (Table S2). In addition, sera collected from mice immunized with a recombinant EIII protein derived from the NGC were capable to recognize the protein found in JHA1 (Figure 5). As indicated in Figures 5A and 5B, sera of sham-treated mice did not recognize the viral particles inside infected cells, while anti-EIII antibodies, raised in mice immunized with a recombinant protein derived from the NGC strain, reacted with the JHA1 particles (Figures 5C and 5D). Moreover, the same anti-EIII serum was shown to neutralize the JHA1 strain, as demonstrated in virus-neutralization assays (Figure 5E). Considering similar cross-reactivity of the NS1-specific antibodies (Figure 4C), these evidences indicate that the JHA1 and NGC strains share important immunological determinants both in the main envelope glycoprotein and the NS1 protein.

## Discussion

The incidence of DF and DHF/DSS cases has dramatically increased during the last decade in different parts of the tropical world and particularly among Latin America countries, in which the disease has assumed a seasonal epidemic character with remarkable numbers of infected subjects and fatalities [42], [43]. This scenario is worsened by the fact that the viral pathogenicity, particularly regarding aspects dictating the infection course and the onset of more serious symptoms, has not been elucidated. This knowledge gap is attributed at least in part to the lack of adequate experimental models that reproduce the pathological and immunological events either leading to the disease or controlling it. In the present study, we described the characterization of a DENV2 strain, named JHA1, which is naturally lethal to mice. After isolation from a symptomatic patient, a virus seed stock was obtained after a minimal number of replication rounds and demonstrated to replicate, induce symptoms and kill Balb/c mice infected via the i.c. administration route. The exacerbate murine virulence of the JHA1 strain probably lies in the natural genetic polymorphism of the virus, in which at least three amino acid replacements were previously ascribed to DENV2 neurovirulence in mice. JHA1-infected mice showed altered hematological parameters similar to those observed in severe dengue cases, such as plasma leakage and hemorrhage. The virus showed rather low infective doses (LD_50_ of 50 PFU) after administration via the i.c. route and, in addition, induced encephalitis, acute inflammatory responses and early antibody responses to the NS1 and, to a lesser extent, the envelope glycoprotein, in agreement with the pathological signs detected among infected subjects. Together, the present findings indicate that the JHA1 strain represents a new and useful tool for the study of DENV pathogenicity and yields promising perspectives regarding the development of a more rational and effective screening program for drugs and vaccine candidates.

A phylogenetic analysis grouped the JHA1 strain as a type 2 DENV (DENV2) strain clustered within strains of the American genotype. Interestingly, representative viruses of this group were isolated in India, American Samoa and Indonesia, which is explained by the transmission of DENV2 strains between Asia and the Americas sometime around 1948 [44], [45]. The DENV2 JHA1 strain was also shown to carry important genetic markers associated with neurovirulence in mice when compared with strains of the same genotype. Two amino acid replacements, D390N and F402L, in the E protein were previously found to be related to neurovirulence in mice [20]. In addition, the replacement at position R105Q of the NS1 protein was also found to be related to neurotropism in mice and is identical or equivalent to those replacements observed in the DENV2 NGC strain [20]. Other amino acid replacements, specifically located at the EIII (positions 301, 303 and 363), were found only in the JHA1 strain. Although not yet ascribed to murine neurovirulence, mutations in this domain that are directly involved in binding host cell receptors may affect host-virus interactions [46]–[48]. The higher virulence of the JHA1 strain compared with the NGC strain, as inferred both by the lower LD_50_ and the lack of morbidity signs among animals who survived a sublethal dose [22], [35], [49], [50], may also be attributed to the unique amino acid replacement detected in the E/NS1 protein sequence as well as in other protein sequences of the virus.

The JHA1 strain shares immunological determinants with the reference DENV2 NGC strain, as demonstrated by the specific reactivity of anti-EIII and anti-NS1 antibodies. To date, the NGC strain has been frequently used in studies employing immunocompetent mice and as the genetic background for the construction of chimeric-, DNA- and protein-based DENV2 vaccine candidates [4], [16], [22], [35], [50], [51]. The present data demonstrated that the JHA1 strain may represent a complementary tool for the evaluation of NGC-based vaccine candidates. In addition, our results indicate that the JHA1 strain may also be used in experimental models without the need to determine how to handle a virus strain in which different mutations have been selected during adaptation to the new host, which is therefore different from the original virus strains.

Pathological damage caused by the JHA1 strain was mainly restricted to the brain and local endothelial cells. In contrast to DHS/DHF cases [43], [52], no significant pathological damage was detected in the liver or spleen of the infected animals. This indicates that the cellular targets of the JHA1 strain are mainly restricted to the cerebral tissue, leading to death by encephalitis. Such pathological features may restrict the dissemination of the virus to other tissues, as supported by the unsuccessful attempts to infect mice with the JHA1 strain using other administration routes (unpublished observations). Nevertheless, the detection of viable virus particles in the spleen and the induction of rather early virus-specific antibody responses indicate that at least some virus particles leak from the cerebral tissue and may reach distant organs. The presence of viable virus particles in the spleen may also be a consequence of the high concentration of mononuclear cells in this organ, known to be permissive to DENV replication [1], [2], [14]. Some of the features detected in mice lethally infected with the JHA1 strain have also been observed in infected subjects with unusual DENV infection manifestations, who suffer from pain ascribed to transient encephalitis and weight loss [53]–[56]. Other DENV experimental models based on immunocompromised mice could also reproduce some of these symptoms (encephalitis and weight loss) but in a different time period [25]. The fact that the JHA1 virus could replicate and kill immunocompetent mice without accumulation of selective mutations allowing adaptation to the new host emphasizes the fact that the natural genetic variability of the virus may hinder relevant information regarding host specificity and tissue tropism among immunocompetent hosts.

Specific hematological alterations observed in JHA1-infected mice were similar to those recorded in severe DENV infection cases, such as the transient increase in hematocrit values followed by a sharp decrease indicative of hemorrhage [25], [54], [57], [58]. In addition, the observed reduction in the number of lymphocytes and neutrophils has also been recorded in DHF and DSS cases in which the depletion of progenitor cells occurs due to medullar virus replication [34], [59]–[62]. In contrast, no alteration was observed in the platelet number during the infection course, which is a hallmark DHF characteristic. A similar feature was also reported for a DENV3 strain capable of infecting immunocompetent mice [26]. However, the coagulation function in mice infected with the JHA1 strain was drastically altered, as measured by the prothrombin time test. This result has a close relationship with the hemorrhage process observed in the JHA1-infected animals and may also be related to the high levels of anti-NS1 antibodies detected in these mice, once that such antibodies were previously shown to interfere with coagulation mechanisms [4], [6], [7]. Nevertheless, the conclusion that the JHA1 strain reproduces, under experimental conditions, most of the hematological disturbances observed in severe forms of DENV infection cases must be considered in future studies that are designed to provide a better understanding of viral pathogenicity.

The JHA1 strain was also shown to induce acute inflammatory reactions and early adaptive immune responses in infected mice. Infected animals had increased serum levels of INF-γ, an important antiviral cytokine that is lacking in AG129 mice (lacking interferon-α/β and -γ receptors), which are frequently used as a DENV infection model [24], [25] Thus, the lack of detectable virus particles in blood samples and histological injuries in organs other than the brain of mice infected with the JHA1 strain may be attributed to the early virus replication control triggered by the rapid increase in INF-γ in immunocompetent Balb/c mice. Similar to the response detected in humans [63], early antibody responses detected in mice infected with the JHA1 strain were mainly associated with the NS1 protein. These results are indicative of the higher immunogenicity of the NS1 protein compared with the E protein and reflect the early synthesis and secretion of the NS1 protein during the infection course [43], [63]. Together, these findings indicate that in mice, the JHA1 strain can reproduce some of the inflammatory reactions and early antibody responses that are detected in DENV-infected subjects without the need to use genetically modified mice. Such features open the possibility of using the JHA1 strain to evaluate immunization regimens that induce protective innate and adaptive immune responses leading to protection from lethal virus challenges.

It is important to highlight that the results presented in this study were obtained by using an inbred immunocompetent mouse lineage, which differ from conditions naturally found among human populations. Probably some of the reported symptoms and features observed in JHA1-infected mouse may differ from those observed in outbred animals. Nonetheless, all experimental murine models aiming to the study of dengue pathogenesis are based on isogenic lineages [6], [26], [64]. In addition, the infection route presented in this study is clearly far from that observed in the infection natural course, with disease symptoms based mainly on encephalitic manifestations, which are accepted as unusual manifestations in infected humans [53]–[56], but in contrast, were also reported in other mouse models with DENV inoculation through the intracranial route [65]–[67]. Thus, the infection model presented here is not able to reproduce the complete set of symptoms seen in DF or its more severe forms, DHF and DSS. Nonetheless, we report here, for the first time, the contribution of reproducing most of the hematological disturbances seen in infected humans by using non-modified virus and mice. Despite the important differences regarding the natural infection, the present model represents a step forward in the study of different aspects of DENV pathogenesis and may help of the screening of different anti-viral approaches.

In conclusion, the present study provides important contributions to the study of DENV2 pathogenicity that may aid the development of antivirus prophylactic and therapeutic approaches. As previously observed [26], our results demonstrate that the endogenous DENV2 genetic plasticity represents an important source of information that may contribute to a better understanding of viral pathogenicity, including the ability of viruses to replicate in a murine host and the severity of the induced symptoms. These results also indicate that a similar approach may be applied to other DENV types that could lead to the identification of virus strains with more adequate features for the development of alternative infection models.

## Supporting Information

Figure S1 **Multiple sequence alignment of the amino acid sequences corresponding to the EIII/NS1 region of the JHA1, India/1957, Indonesia 1977 and NGC strains.** Polymorphic sites are indicated and placed into three different regions of the analyzed sequences: domain III (red), stem-anchor and NS1 signal peptide (blue) of the E protein (inside the black rectangle) and the entire NS1 protein (outside of the black rectangle).
(TIF)

Table S1 **Comparison of amino acid sequences of the envelope glycoprotein and nonstructural NS1 protein regions possibly involved with neurovirulence in mice of the NGC and JHA1 DEN2 strains.**
(DOC)

Table S2 **Predicted T-cell epitopes shared by the JHA1 and NGC DENV2 strains found at the sequences encoding the EIII/NS1 proteins.** ^a^ Inferred amino acid sequences of NGC and JHA1 strains were submitted to the computational system PRED^BALB/C^ to predict specific epitopes for class I (H2-K^d^, H2-L^d^ and H2-D^d^) and class II (H2-IE^d^ and H2-IA^d^) MHC molecules of Balb/c mice (Zhang et al., 2005). The predicted epitopes with higher scores were compared between the two strains to infer the conservation of these immunological determinants. This comparison was also applied to the experimentally determined CD8^+^ T cell-restricted epitope of the DENV2 NS1 protein, AGPWHLGKL (Gao et al., 2008). ^b^ Predicted epitopes with higher scores within the EIII/NS1 region of the JHA1 isolate and the CD8^+^ T cell-restricted epitope of the DENV2 NS1 protein, all of which were fully conserved between the JHA1 and NGC strains. ^c^ Location of the conserved epitope within the EIII/NS1 region of the strains subjected to the analysis. ^d^ Epitope located in the NS1 protein previously demonstrated to be specific for CD8^+^ T lymphocytes and widely conserved among several DENV2 strains.
(DOC)
