# Supplementary figures and images for: A Genetic and Pathologic Study of a DENV2 Clinical Isolate Capable of Inducing Encephalitis and Hematological Disturbances in Immunocompetent Mice
Source: PLoS One. 2012 Sep 13;7(9):e44984. doi: 10.1371/journal.pone.0044984 (PMC3441697; doi:10.1371/journal.pone.0044984)

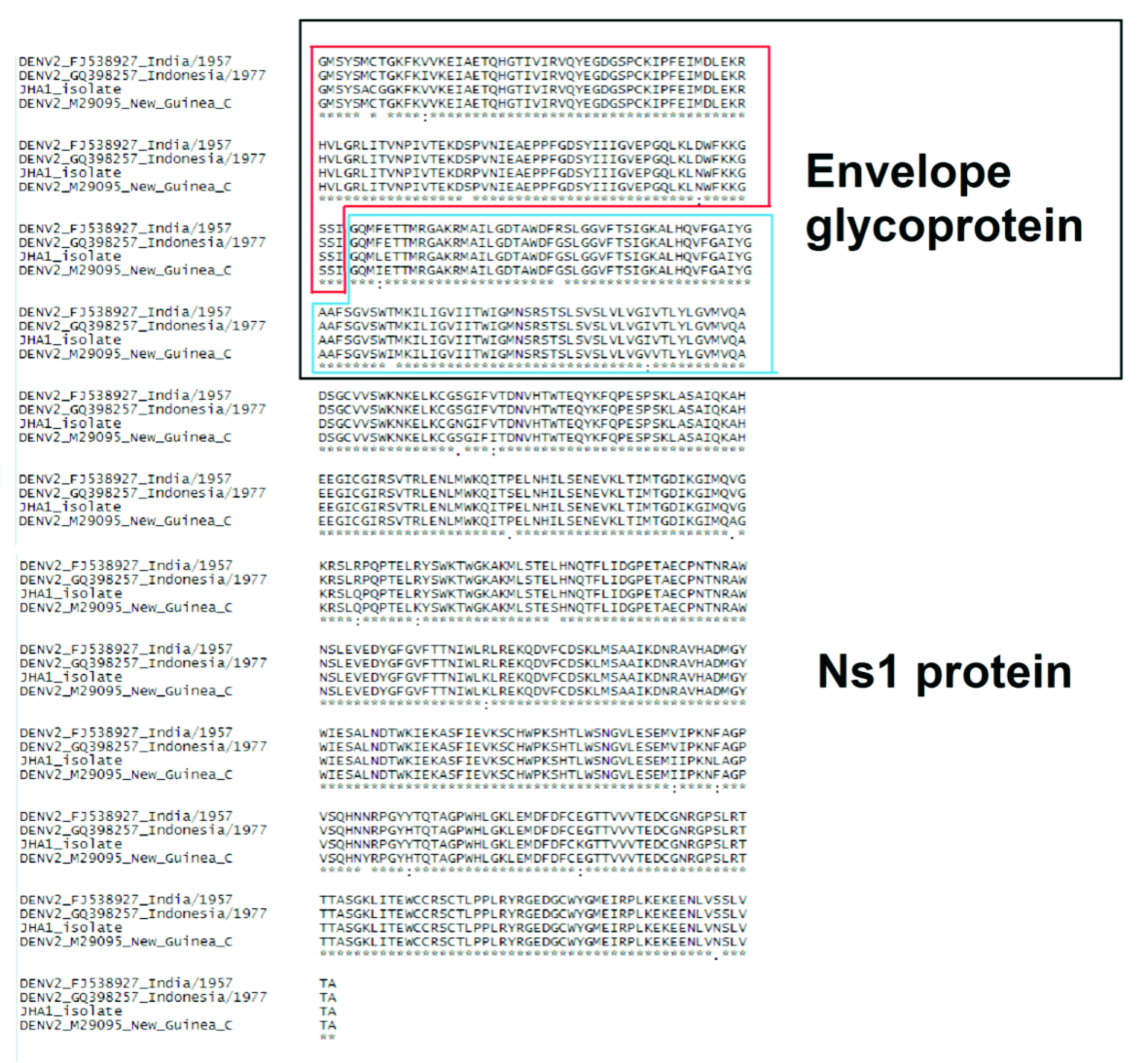

Supplement: Figure S1 — Multiple sequence alignment of the amino acid sequences corresponding to the EIII/NS1 region of the JHA1, India/1957, Indonesia 1977 and NGC strains. Polymorphic sites are indicated and placed into three different regions of the analyzed sequences: domain III (red), stem-anchor and NS1 signal peptide (blue) of the E protein (inside the black rectangle) and the entire NS1 protein (outside of the black rectangle). (TIF) [file pone.0044984.s001.tif]
