# Supplementary material for: A Genetic and Pathologic Study of a DENV2 Clinical Isolate Capable of Inducing Encephalitis and Hematological Disturbances in Immunocompetent Mice
Source: PLoS One. 2012 Sep 13;7(9):e44984. doi: 10.1371/journal.pone.0044984 (PMC3441697; doi:10.1371/journal.pone.0044984)
Supplement: Table S1 — Comparison of amino acid sequences of the envelope glycoprotein and nonstructural NS1 protein regions possibly involved with neurovirulence in mice of the NGC and JHA1 DEN2 strains. (DOC) [file pone.0044984.s002.doc]

**Table S1**

|  | Amino acid | |
| --- | --- | --- |
| Protein and position | NGC | JHA1 |
| EIII (301) | M | A |
| EIII (303) | T | G |
| EIII (363) | S | R |
| EIII (390) | N | N |
| E stem-anchor (402) | I | L |
| E stem-anchor (454) | I | T |
| NS1 (105) | Q | Q |
