# Supplementary material for: A Genetic and Pathologic Study of a DENV2 Clinical Isolate Capable of Inducing Encephalitis and Hematological Disturbances in Immunocompetent Mice
Source: PLoS One. 2012 Sep 13;7(9):e44984. doi: 10.1371/journal.pone.0044984 (PMC3441697; doi:10.1371/journal.pone.0044984)
Supplement: Table S2 — Predicted T-cell epitopes shared by the JHA1 and NGC DENV2 strains found at the sequences encoding the EIII/NS1 proteins. a Inferred amino acid sequences of NGC and JHA1 strains were submitted to the computational system PREDBALB/C to predict specific epitopes for class I (H2-Kd, H2-Ld and H2-Dd) and class II (H2-IEd and H2-IAd) MHC molecules of Balb/c mice (Zhang et al., 2005). The predicted epitopes with higher scores were compared between the two strains to infer the conservation of these immunological determinants. This comparison was also applied to the experimentally determined CD8+ T cell-restricted epitope of the DENV2 NS1 protein, AGPWHLGKL (Gao et al., 2008). b Predicted epitopes with higher scores within the EIII/NS1 region of the JHA1 isolate and the CD8+ T cell-restricted epitope of the DENV2 NS1 protein, all of which were fully conserved between the JHA1 and NGC strains. c Location of the conserved epitope within the EIII/NS1 region of the strains subjected to the analysis. d Epitope located in the NS1 protein previously demonstrated to be specific for CD8+ T lymphocytes and widely conserved among several DENV2 strains. (DOC) [file pone.0044984.s003.doc]

**Table S2**

| **Balb/C mouse MHC molecule or T cell^a^** | **Predicted or validated epitope^b^** | **Location^c^** |
| --- | --- | --- |
| H2-K^d^ | SLGGVFTSI | Stem-anchor region of the E protein |
| H2-L^d^ | RSVTRLENL | NS1 protein |
| H2-D^d^ | SKLMSAAIK | NS1 protein |
| H2-IE^d^ | FKVVKEIAETQHGTI | Domain III of the E protein |
| H2-IA^d^ | PETAECPNTNRAWNS | NS1 protein |
| TCD8^+^ (Gao et al., 2008) | AGPWHLGKL**^d^** | NS1 protein |
